# Managing Toe Walking, a Treatment Side Effect, in a Child With T-Cell Non-Hodgkin's Lymphoma: A Case Report

**DOI:** 10.3389/fped.2019.00502

**Published:** 2019-12-13

**Authors:** Wojciech Kiebzak, Arkadiusz Żurawski, Anna Zmyślna, Justyna Pogorzelska, Małgorzata Domagalska-Szopa, Magdalena Hagner-Derengowska, Andrzej Szopa

**Affiliations:** ^1^Department of Physiotherapy, Faculty of Medicine and Health Sciences, Jan Kochanowski University, Kielce, Poland; ^2^Centre for Pediatrics, Regional Hospital in Kielce, Kielce, Poland; ^3^Department of Medical Rehabilitation, School of Health Sciences in Katowice, Medical University of Silesia, Katowice, Poland; ^4^Faculty of Earth Sciences and Spatial Management, University Nicolaus Copernicus, Torun, Poland; ^5^Department of Physiotherapy, School of Health Sciences in Katowice, Medical University of Silesia, Katowice, Poland; ^6^Medical Rehabilitation Center Neuromed SC, Katowice, Poland

**Keywords:** childhood cancer, toe walking, DIERS formetric 4D, physiotherapy, gait disorders

## Abstract

**Background and Purpose:** Children who have survived cancer are at risk of experiencing adverse effects of the cancer or its treatments. One of the adverse effects may be the limitation of ankle dorsiflexion (DF), which may result in “toe walking.” Although there is an increasing number of studies in pediatric oncology presenting evidences of different therapeutic interventions to improve DF function, none of these therapeutic interventions has been sufficiently documented. This case report shows the results of non-invasive neurodevelopmental treatment program combined with application of inhibiting casts in a pediatric cancer patient who presented with severe and persistent toe walking. The treatment was aimed to improve DF function and postural and gait pattern and to normalize weight distribution between forefoot and heel.

**Case Presentation:** A 7-year-old girl with T-cell lymphoma, who presented with severe and persistent toe walking, was assessed 10 times over a course of 6 months by both clinical examination (ankle range of motion measurement) and neurophysiological measures (weight distribution between forefoot and hindfoot, postural sway, body posture, and gait).

**Outcomes:** Neurodevelopmental treatment program combined with application of inhibiting casts for 3 months increased passive ankle DF by 10° in both lower limbs, normalized weight distribution between the forefoot and heel in both lower limbs, as well as established a heel-toe walking gait pattern. Improved ankle DF function and normalized postural and gait patterns were maintained in repeated examinations even 3 months after the removal inhibiting casts.

**Discussion:** Early identification of toe walking in the female pediatric patient with T-cell non-Hodgkin's lymphoma and early physiotherapy intervention were beneficial in terms of her body posture and gait pattern development. Non-invasive neurodevelopmental treatment program combined with application of inhibiting casts as described in this study can be useful for managing treatment side effects in pediatric cancer patients.

## Background and Purpose

Non-Hodgkin's lymphomas (NHLs) constitute a heterogeneous group of lymphomas affecting lymphoid tissue in persons of all ages and account for 10% of aggressive lymphomas in the world (1). According to the World Health Organization (WHO), NHL are classified as either B- or T-cell lymphomas (T-NHL) (2). Patients with T-NHL are characterized by a variety of clinical manifestations, including systemic symptoms (fever above 38°C, over 10% weight loss, and severe night sweats), hepatosplenomegaly, generalized lymphadenopathy, and often various skin, spleen, bone marrow, and blood disorders (2, 3). According to many published studies, lymphoid malignancies developed form T lymphocytes are generally more aggressive and show a much worse prognosis than do B-cell lymphomas (4, 5).

The most frequently listed specific symptoms of T-NHL include painless swelling in lymph nodes and constitutional symptoms (2, 3). Most of the patients with T-NHL are treated with conventional chemotherapy, which consists of cyclophosphamide, doxorubicin, vincristine, and prednisone. Studies in pediatric oncology agree that both the lymphomas as well as the cancer therapy can produce adverse effects (6, 7). Especially, children with T-NHL are at a high risk of experiencing adverse effects of the cancer or its medical treatments (6, 7). One of the adverse effects may be limitation of ankle dorsiflexion (DF), which may result in “toe walking” (TW). Although there is an increasing number of studies in pediatric oncology presenting evidences of different therapeutic interventions to improve DF function, none of them has been sufficiently documented (8, 9).

The aim of this study was to assess the outcomes of a non-invasive neurodevelopmental treatment program combined with application of inhibiting casts in a 7-year-old girl with T-NHL, who had previously received cancer treatment and then developed TW.

## Case Presentation

A 7-year-old girl presented with severe and persistent TW after cancer therapy and was clinically followed up for 6 months (parental consent was obtained).

When she was 6 years and 3 months old, she was hospitalized at the Department of Oncology and Haematology, Centre of Paediatrics, Kielce, Poland with a 3-month history of generalized lymphadenopathy, weight loss, night sweats, and intermittent low-grade fever. Based on the result of the biopsy of the mediastinal tumor, the diagnosis of mediastinal T-NHL (according to International Classification of Diseases 10: C83.5) was confirmed. Then, she was admitted to the Department of Chemotherapy of the Centre of Paediatrics, where she was treated in line with the EURO-LB 02 protocol for 3 months.

Pediatric prednisone and intrathecal methotrexate were given on the first and seventh day of treatment, and then, the patient received prednisone, followed by 6-mercaptopurine and four cycles of methotrexate. Following stratification, the patient was assigned to stage I, which translated into a maintenance therapy consisting of 6-mercaptopurine once a day and methotrexate once a week.

Inpatient stays of 3 months and treatment-related immobility and anxiety of physical activity caused muscle weakness in her lower limbs and limited her mobility. Postdischarge from the hospital, the child gradually began to increase the walking distance, but she walked on her toes more often. One month after chemotherapy completion, the parents estimated that the patient walked on her toes 100% of the time. Examination of a cerebrospinal fluid sample revealed neither any blasts nor any evidence of neurological complications; therefore, the patient was referred to physiotherapy.

Severe and persistent TW, i.e., the failure of the heel to make contact with the floor at the onset of stance and absence of a heel rocker, could be observed in the patient (10). Clinical evaluation of body posture in the upright position revealed misorientation of body segments, such as excessive backward pelvic tilt, excessive kyphosis, asymmetric postural alignment in the frontal plane with s-shaped scoliosis, and excessive pelvic obliquity and rotation on the left side resulting in the lack of a flat right foot contact.

In addition, clinical assessment of passive range of motion (ROM) of ankle joint revealed a large deficit in ankle DF-ROM, defined as the value obtained from the neutral foot position in both lower limbs.

The attempts to facilitate physiological pressure on the feet while standing were successful only when both the patient and observer were constantly monitoring the process. Squatting was possible only with the patient holding on to a support or when being supported by another person (and with assistance to lift the heels 10 cm above the ground level).

### Intervention

Non-invasive neurodevelopmental treatment program including neurophysiological therapy based on the Vojta approach, combined with application of inhibiting casts, was carried out for 3 months. The aim of the treatment was to improve DF function and postural and gait pattern and to normalize weight distribution between forefoot and heel.

The patient completed two physiotherapy sessions per week for the first 3 months and then one session per week until the end of her clinical follow-up. All the sessions took place at the rehabilitation clinic. The complementary treatment consisted of the using the inhibiting casts for 3 weeks in the second month of the entire therapeutic program.

Bilateral short below-the-knee casts were applied with the patient in the supine position with the hip and knee flexed at 90°, and the leg and foot rested in the neutral position (10, 11). After removing the casts, an adjunctive therapy using formthotics insoles was introduced for active foot posture correction.

### Examination

All study protocols were duly approved by the Local Bioethics Committee. The patient was clinically and functionally monitored for 6 months by (1) measuring ankle passive ROM of both legs, (2) evaluating body weight distribution between forefoot and hindfoot of both lower limbs, (3) measuring postural sway based on center-of-foot pressure (CoP) movement, (4) analyzing body posture based on stereography, and (5) analyzing gait.

Complete examination was performed before and after the treatment (pre- and posttreatment, respectively; 1st and 10th examination), before and after the application of inhibiting casts (pre- and postcasting, respectively; 5th and 6th examination), three times between pretreatment and precasting examination (2nd, 3rd, and 4th examination), three times between postcasting and posttreatment examination (7th, 8th, and 9th examination), and 1 month after the treatment (11th examination).

The first step of examination was to assess passive ROM values of ankle joints separately for each lower limb using an accelerometer-based system (12).

Analyses of static load (WB) distribution, postural sway based on CoP movement, surface topography of body posture, and gait were carried out using DIERS Formetric 4D system (DIERS International GmbH, Germany), a light-optical scanning method based on video raster stereography (13–16). The system projects a line grid on the back of the patient, recorded by an imaging unit. A computer software analyzes the line curvature and generates a three-dimensional model of the body surface using photogrammetry (13–16).

Combined usage of DIERS formetric 4D and DIERS pedoscan system allowed simultaneous measurements of body posture, including the spinal form (e.g., frontal and lateral spinal curvature, vertebral rotation), 3D pelvic position, static body weight distribution, and CoP movements, possible. For the examination of body posture, it was necessary to uncover the back surface of patient and manually place reflective markers on the spinous process of C7 and bilateral posterior superior iliac spines. The examination was performed in the standing position. The participant stood barefoot on a pedographic platform (situated on the ground) in a natural position (feet abducted at 20°, heels 3 cm apart) with the arms hanging loosely at the sides. Each measurement was recorded three times (each trial lasting for 30 s with a 30-s pause between two trials). The mean values from three trials were used to calculate the postural parameters, WB distribution, and stability indices from the CoP trajectories. To characterize the dynamic and static body posture a set of parameters were chosen (Table 1) (15, 16).

**Table 1:** Definitions of the selected postural indices, weight-bearing distribution on the support base, posturographic CoP shifts, and gait parameters obtained using DIERS Formetric 4D system for monitoring patient's improvement.

**CoP shifts (SP)**	**(cm)**	**Sway path length of the CoP**
Angle of kyphosis (KK)	(°)	The angle between the spinous process of C7 and the thoracic-lumbar inflection point
Angle of lordosis (LL)	(°)	The angle between the thoracic-lumbar inflection point and the midpoint between the two lumbar dimples
Trunk inclination (TI)	(mm)	The angle between the midpoint between the two lumbar dimples and the spinous process of C7
Pelvic tilt (PT)	(°)	The angle between the line connecting C7 and S1 and the line connecting *L*max and S1
Trunk surface rotation (TSR)	(°)	The angle of trunk surface rotation is contained between the line situated in the frontal plane and the line which connects two points on the back surface and is situated symmetrically on the left and right sides
LF	(%)	Percentage of load distribution for the left forefoot
RF	(%)	Percentage of load distribution for the right forefoot
LH	(%)	Percentage of load distribution for the left hindfoot
RH	(%)	Percentage of load distribution for the right hindfoot
Stance time	(% gait cycle)	Duration of the stance phase, expressed as the percentage of the gait cycle
Load response time	(% gait cycle)	Duration of load response phase, expressed as the percentage of the gait cycle
Single support	(% gait cycle)	Duration of single support phase, expressed as the percentage of the gait cycle
Preswing time	(% gait cycle)	Duration of preswing phase, expressed as the percentage of the gait cycle
Swing time (SwT)	(% gait cycle)	Duration of swing phase, expressed as the percentage of the gait cycle
Step time (Step)	(s)	Time interval between two successive instants of foot–floor contact during walking
Stride width (Stride)	(cm)	Distance between two lines dividing the feet into equal halves

The gait data were recorded as the subject walked 16 m without shoes and assistive devices on a treadmill (DIERS pedogait, DIERS International GmbH, Germany) with integrated pressure plate at a speed of 2 km/h. The mean values from all gait cycles for the distance of 16 m were used to calculate a set of spatiotemporal and kinetic gait parameters (Table 1).

All the above-mentioned values were automatically registered using a dedicated software. The indices presented in Table 1 were chosen for future analysis.

Statistical significance of the obtained results was determined using *Z* test, a test to determine the mean value of the population from which a sample was collected (m, sample mean; S, sample SD). The *p*-value indicated the level of statistical significance for the differences between the last measurement (11th examination) and all the previous measurements (1–10th examinations). Software Statistica 12 Pl Stat Soft was used to statistical analysis.

### Outcomes

After 3 weeks of treatment (precasting examination), the subject showed only a slight increase in the passive ROM of ankle DF. After cast removal (postcasting examination), ankle DF ROM immediately increased and was around 15° in left and 10° in right limb throughout the treatment until the last examination after treatment. The normal range for ankle joint dorsiflexion is 10° to 30°.

**Figure 1:**
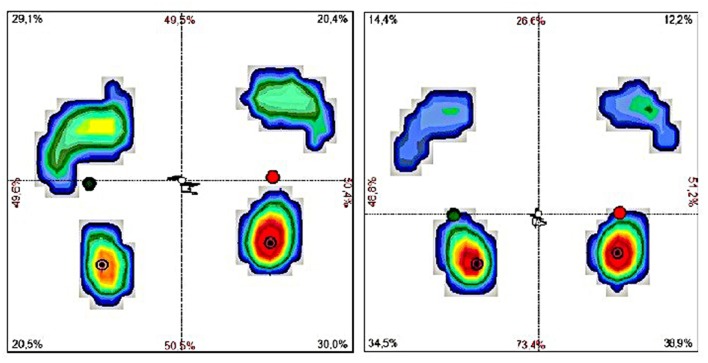
The results of examination of body weight distribution between forefoot and hindfoot of both lower limbs and postural sway measurement based on CoP movement in pre- (right) and posttreatment (left) examination based on the assessment using DIERS pedoscan system.

Simultaneously, the normalization of body weight distribution between forefoot and hindfoot of both lower limbs was achieved. Compared to the posttreatment weight distribution between forefoot and hindfoot of both limbs, pretreatment static foot pressure measurement showed significant overloading of forefoot of both legs. The forefoot pressure significantly decreased from 29.13 to 14.36% in the left foot (*p* = 0.002) and from 20.38 to 12.24% in the right foot (*p* = 0.006) and became close to the normal (Figure 1). Posttreatment dynamic measurement also revealed an improvement in body balance, reflected by a reduction in sway of CoP movement from 124.11 cm at baseline to 71.46 cm at final measurement (*p* = 0.017) (Table 2).

**Table 2:** Summary of the postural indices, weight-bearing distribution on the support base, and posturographic CoP shifts all the examinations (1st−11th examination) assessed using DIERS pedoscan.

	**Examination**											***p***
	**1**	**2**	**3**	**4**	**5**	**6**	**7**	**8**	**9**	**10**	**11**	
SP (cm)	124.11	107.4	78.77	77.49	68.58	89.28	74.46	71.92	69.12	73.64	71.46	0.017
KK (°)	28.59	29.3	14.24	24.86	31.03	27.55	28.92	26.38	21.92	31.1	35.73	0.046
LL (°)	46.02	45.05	43.02	38.16	45.14	34.02	40.74	37.72	40.15	40.28	36.88	0.004
PT (°)	30.73	24.67	31.19	25.81	26.55	21.96	26.67	23.02	26.56	25.72	27.58	0.918
TI (°)	8.16	5.78	6.82	3.95	5.57	2.03	1.78	2.47	3.23	2.64	2.91	0.01
LF (%)	29.13	30	17.98	13.6	13.16	19.87	17.76	18.41	15.02	14.62	14.36	0.002
RF (%)	20.38	24.01	10.01	12.38	13.21	12.62	12.94	11.54	14.91	14.14	12.24	0.006
LH (%)	20.46	24.71	38.4	36.99	38.89	33.67	33.35	36.8	33.88	31.64	34.45	0.222
RH (%)	30.08	21.28	33.62	37.03	34.74	33.84	35.95	33.25	36.2	39.6	38.94	0.036

*SP, CoP shifts; KK, angle of kyphosis; LL, angle of lordosis; TI, trunk inclination; PT, pelvic tilt; TSR, trunk surface rotation; LF and RF, left and right forefoot percentage load distribution; LH and RH, left and right hindfoot percentage load distribution*.

**Figure 2:**
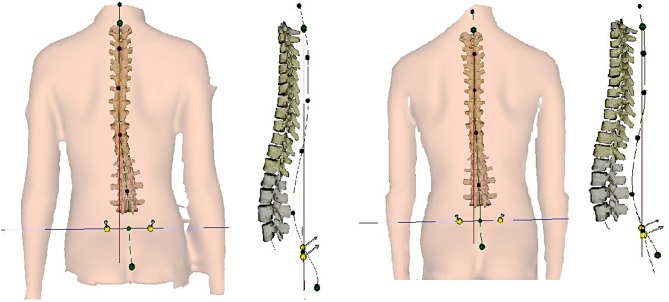
Spinal curvature in the coronal and sagittal plane in pre- (right) and posttreatment (left) examination based on the assessment using DIERS system.

Even 1 month after the completion of treatment, the patient showed improved body segment alignment in all three dimensions, manifested by decrease in trunk forward inclination (from 28.59 to 35.73, *p* = 0.046), thoracic kyphosis angle (from 46.02° to 36.88°; *p* = 0.004), and lumbar lordosis angle and degree of trunk surface rotation (from 8.16° to 2.91°; *p* = 0.01) (Table 2 and Figure 2).

**Figure 3:**
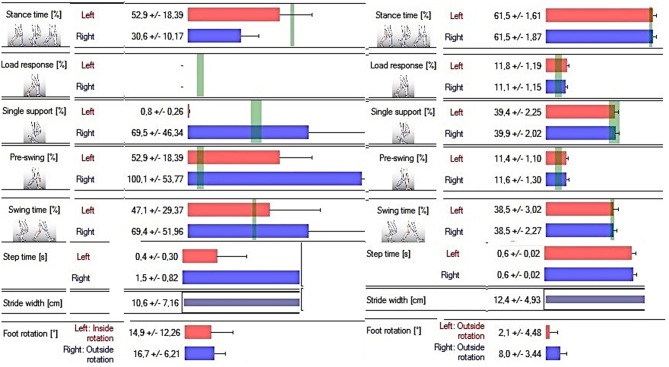
The results of gait analysis based on the pre- and posttreatment examination (respectively; on the right and left) using the DIERS pedoscan system. Green vertical lines indicate the normal values.

Posttreatment gait analysis revealed notable improvement in the gait pattern. Most of the analyzed gait parameters in posttreatment assessment showed values close to the normal values (green vertical lines indicate the normal values) (Figure 3) (17).

## Discussion

Although studies in pediatric oncology confirm the impairment of ankle DF-ROM and/or ankle DF strength in the patients with acute lymphoblastic leukemia and in the survivors/long-term survivors and mixed cancer survivors (7–9, 18), there is a lack of studies on occurrence of TW in the patients with childhood cancer. Recent studies have reported many possible limitations of ankle DF-ROM treatment; however, none of them considered the consequences of the treatment, such as misalignment of body segments or gait disturbances. In addition, none has been sufficiently documented.

As there are no studies clearly explaining the etiology of TW in children during/after cancer treatment, TW has been hypothesized to be caused due to multiple factors. First, the lack of walking due to long-term hospitalization and treatment-related immobility results in limited ankle DF-ROM, which may cause TW. Second, TW may have resulted from limited ankle DF-ROM due to peripheral neuropathy, which is commonly observed in the patients treated for oncological diseases (17, 19). Third, myopathy and atrophy in gastrocnemius muscle have been reported as the adverse effects of certain chemotherapy drugs (18, 19) and may also restrict primary ankle DF function and then TW as a late effect of the chemotherapy.

The normal range for ankle DF (0° to 16° for non-weight-bearing ankle joint and 0° to 30° for weight-bearing ankle joint) is necessary for normal postural and gait patterns (17). One can presume that impaired ankle DF was particularly important for the development of compensatory strategy manifested by both misalignment of body segments and gait disturbances in this case. Especially, the achievement of the near normal range for ankle joint DF (15° for the left and 10° for the right leg) using inhibiting casts was the most important point of the undertaken treatment program. Our analyses showed that the retrieved ankle DF movement effected the normalization of weight bearing on both feet; however, it was not enough for body posture correction and gait pattern improvement. The extended neurodevelopmental treatment program including neurophysiological therapy based on the Vojta approach was necessary to provide a persistent stretch (20) to the gastrocnemius and soleus contractures and to improve the postural and gait pattern.

The case study presented here was unique because of the non-invasive neurodevelopmental treatment program combined with application of inhibiting casts used in a 7-year-old girl with T-NHL, who previously received cancer treatment and was then affected by TW. Using the functional assessment method, we documented the effect of the undertaken treatment program on ankle DF function correction, normalization of weight distribution between forefoot and hindfoot, and improvement of postural and gait pattern.

### Study Limitation

Although this study indicated that neurodevelopmental treatment program combined with application of inhibiting casts is effective for managing treatment side effects, such as TW in the pediatric cancer patient, the observations were based on only one case. Therefore, further randomized trials are required. As the effect of treatment program is usually difficult to distinguish from the effects of disease recovery, further research including a similar patient population are necessary.

## Conclusion

This case description can enhance the recognition of ankle DF function disturbances and occurrence of TW in children during/after cancer treatment, and it can initiate further research programs focused on targeting and evaluating ankle DF function, body posture, and gait in pediatric oncology patients.

## Data Availability Statement

The datasets generated for this study are available on request to the corresponding author.

## Ethics Statement

The studies involving human participants were reviewed and approved by Bioethics Committee of the Medical University of Silesia. Written informed consent to participate in this study was provided by the participants' legal guardian/next of kin.

## Author Contributions

WK, AŻu, AZm, JP, MH-D, MD-S, and AS drafted the manuscript or revised it critically for important intellectual content, provided final approval of the version to be published, and agreed to be accountable for all aspects of the work in ensuring that questions related to the accuracy or integrity of any part of the work were appropriately investigated and resolved.

### Conflict of Interest

The authors declare that the research was conducted in the absence of any commercial or financial relationships that could be construed as a potential conflict of interest.
